# Sites of extranodal involvement are prognostic in patients with stage 1 follicular lymphoma

**DOI:** 10.18632/oncotarget.19240

**Published:** 2017-07-14

**Authors:** Aditi Shastri, Murali Janakiram, Ioannis Mantzaris, Yiting Yu, Jaime S. Londono, Amit K. Verma, Stefan K. Barta

**Affiliations:** ^1^ Division of Hematologic Malignancies, Department of Oncology, Montefiore Medical Center & Albert Einstein College of Medicine, Bronx, NY, USA; ^2^ Department of Biostatistics, Albert Einstein College of Medicine, Bronx, NY, USA; ^3^ Department of Medical Oncology, Fox Chase Cancer Center, Philadelphia, PA, USA

**Keywords:** follicular lymphoma, Surveillance, Epidemiology and End Results database, prognosis, extranodal disease

## Abstract

**Objectives:**

Follicular lymphoma (FL) is the most common indolent B cell lymphoma in the United States and a quarter of patients present with stage I disease. The objective of this study was to examine if primary site of disease influences survival in early stage lymphoma.

**Results:**

The most common extranodal primary sites were the integumentary system (8%), followed by the GI tract (6.4%) and head & neck (5.6%). We stratified patients into a pre-rituximab era (1983-1998) and the rituximab era (1999-2011). In multivariable analysis, integumentary disease was associated with better overall survival (Hazard Ratio [HR], 0.77; Confidence Interval [CI], 0.66-0.9) while primary site FL of the nervous system (HR, 2.40; CI, 1.72-3.38) and the musculoskeletal system (HR, 2.14; CI, 1.44-3.18) were associated with worse overall survival when compared to primary nodal FL. Treatment in the pre-rituximab era, male gender and older age at diagnosis were associated with worse survival.

**Methods:**

We queried the SEER database from 1983 to 2011. We included all adult patients (>18 years) with histologically confirmed stage I FL, active follow-up, and a single primary tumor. A total of 9,865 patients met eligibility criteria, with 2520 (25%) having an extranodal primary site. We classified the primary sites by organ or anatomic location into 11 sites.

**Conclusion:**

Primary site of disease is a prognostic factor for patients with early stage FL and may help identify subsets of patients that could benefit from early, aggressive treatment.

## INTRODUCTION

Follicular lymphoma (FL) is the most common subtype of indolent lymphomas and accounts for 35% of all Non-Hodgkin’s Lymphomas in the United States [1-3]. In approximately 85% of FL patients, the lymphoma cells harbor the pathognomonic translocation t (14; 18)(q32; q21) leading to overexpression of the BCL2 oncogene, an essential step in the pathogenesis of FL [4]. FL is a heterogeneous disease with variable biologic behavior and clinical course. The Follicular Lymphoma International Prognostic Index or FLIPI score is the most commonly used prognostic tool that has been revised and validated in the rituximab era (FLIPI-2)[5, 6]. Notwithstanding the prognostic classification, treatment of follicular lymphoma is guided primarily by the extent of disease involvement [7]. Advanced stage FL (stage III/IV) is considered an incurable but indolent disease for which the decision to treat is based on several well-established clinical criteria. In contrast, treatment of limited stage FL (stage I and contiguous stage II) has the potential to result in long-term disease-free survival, however the treatment modality of choice is not well defined [7, 8].

Several consensus based practice guidelines recommend radiation therapy (RT) as the preferred treatment for stage 1 follicular lymphoma, which represents approximately 25% of the presenting cases of FL [9, 10]. However, the optimal radiation dose and field size have not been definitively determined to date [10-12] An analysis of the National Lymphocare Study, a large prospective cohort study of patients with FL, revealed that only 27% of the patients with early stage disease were treated with RT while others were only observed, received single-agent rituximab, or a combination of rituximab with chemotherapy with or without subsequent radiotherapy [10]. These practices likely reflect uncertainty over the most beneficial initial treatment strategy coupled with concern about the short and long-term treatment related toxicities of RT and/or chemoimmunotherapy. Evidence suggests that the site of involvement is associated with outcome in several Non-Hodgkin’s lymphomas (NHL’s)[13-17]. Therefore identifying subsets of patients with pathobiologically different tumor types could help tailor initial therapy employed in managing limited stage follicular lymphoma. The objective of this study was to assess how the primary site of involvement influences prognosis and can inform therapy in stage I follicular lymphoma.

## RESULTS

### Patient characteristics

**Figure 1:**
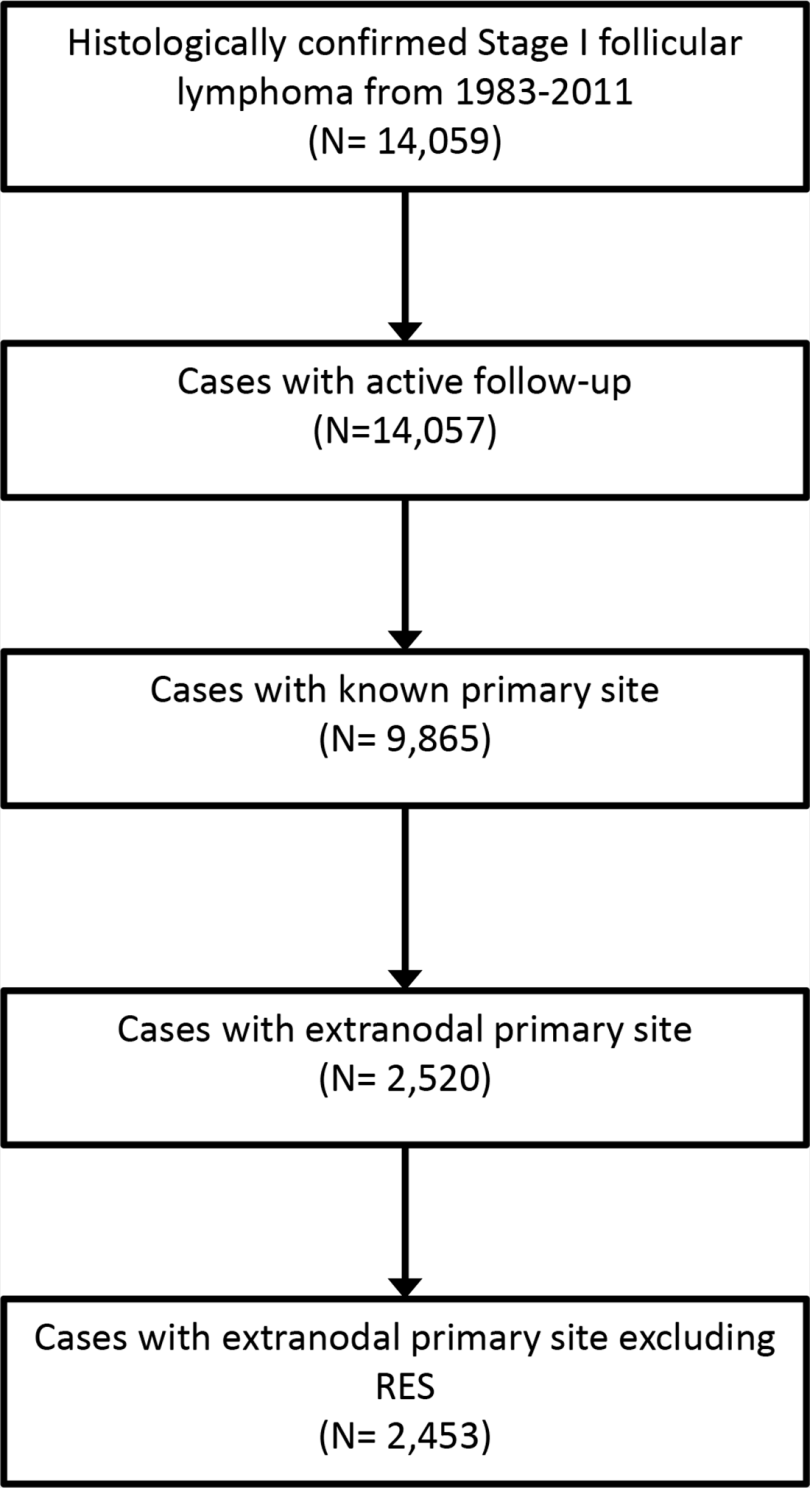
Flowchart showing selection of patients RES, reticuloendothelial system.

**Figure 2:**
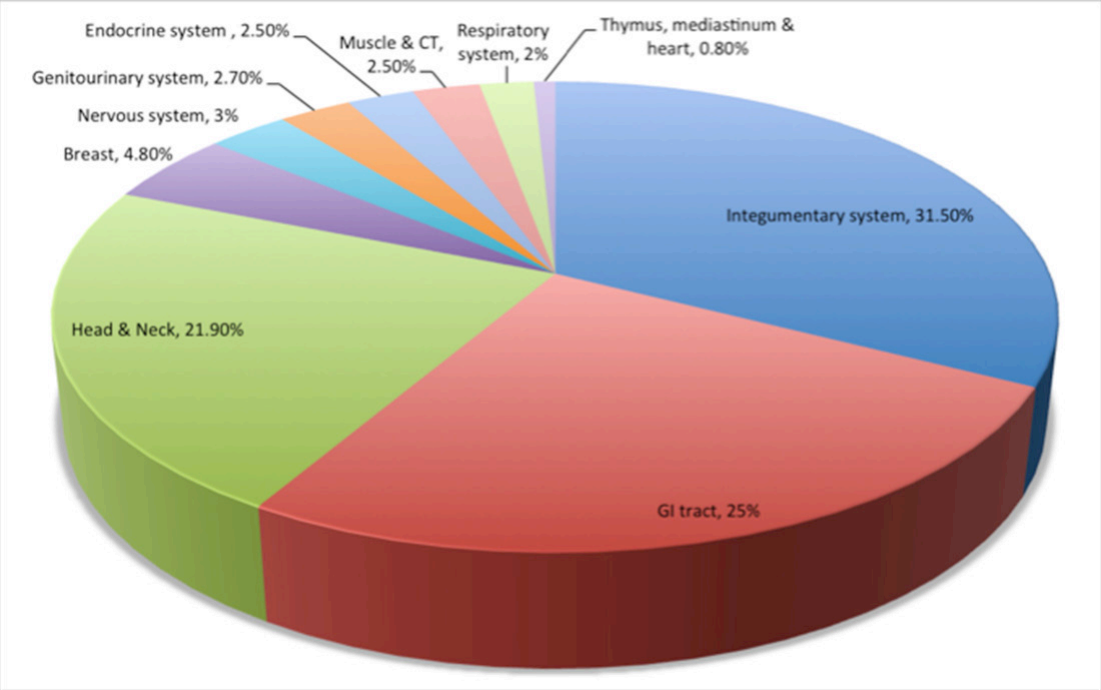
Primary extranodal sites of involvement CT, cardiothoracic; GI: gastrointestinal. *Cases not included here are those that have a known but unclassified primary site and reticuloendothelial system as a primary site.

We identified 14,059 adult cases of histologically confirmed Stage I follicular lymphoma from 1983-2011, of whom 9,865 patients met eligibility criteria (Figure 1). Extranodal primary site was noted in 2,520 patients (25.5%). Patient characteristics are outlined in Table 1. Briefly, of the patients analyzed, 4,749 (48%) were male, 8,833 (90%) were white and 5,459 (55%) were older than age 60. The median age in our series was similar between patients presenting with primary LN disease versus extranodal disease (61.5 vs. 61.8 years). The most common extranodal primary sites were the integumentary system (31.5%), followed by GI tract (25%), Head & Neck (21.9%) and Breast (4.8%; Figure 2).

**Table 1:** Baseline characteristics and comparison within and across primary site categories^1^

Primary site	Total no.	Age >60 (years)	Males	White	Black	Other + unknown*
Lymph Node	7,411 (75)	4,031 (54)	3,572 (48)	6,718 (91)	303 (4)	390 (5)
Integumentary system	793 (8)	412 (52)	438 (55)	703 (89)	43 (5)	46 (6)
GI tract	632 (6)	383 (61)	310 (49)	564 (89)	18 (3)	49 (8)
Head & Neck	552 (5)	341 (62)	264 (48)	480 (87)	28 (5)	44 (7)
Breast	123 (1)	91 (74)	5 (4)	109 (89)	7 (6)	7 (6)
Nervous system	77 (0.7)	41 (53)	41 (53)	67 (87)	4 (5)	6 (7)
Genitourinary system	69 (0.7)	37 (54)	31 (45)	58 (84)	9 (13)	2 (3)
Endocrine system	63 (0.6)	31 (49)	12 (19)	59 (94)	0 (0)	3 (5)
Muscle & CT	62 (0.6)	36 (58)	37 (60)	59 (95)	2 (3)	1 (2)
Respiratory system	62 (0.6)	43 (69)	28 (45)	57 (92)	2 (3)	3 (5)
Thymus, mediastinum & heart	20 (0.2)	13 (65)	11 (55)	18 (90)	1 (5)	1 (5)
**Total**	**9,865**	**5,459 (55)**	**4,749 (48)**	**8,833 (90)**	**417 (4)**	**552 (6)**

CT, cardiothoracic; GI, gastrointestinal.

^1^Data are presented as number (%) unless otherwise indicated.

*Other represents race recodes for American Indian, AK Native, Asian & Pacific Islander; Unknown represents race recode for unknown race.

### Survival analysis

**Figure 3:**
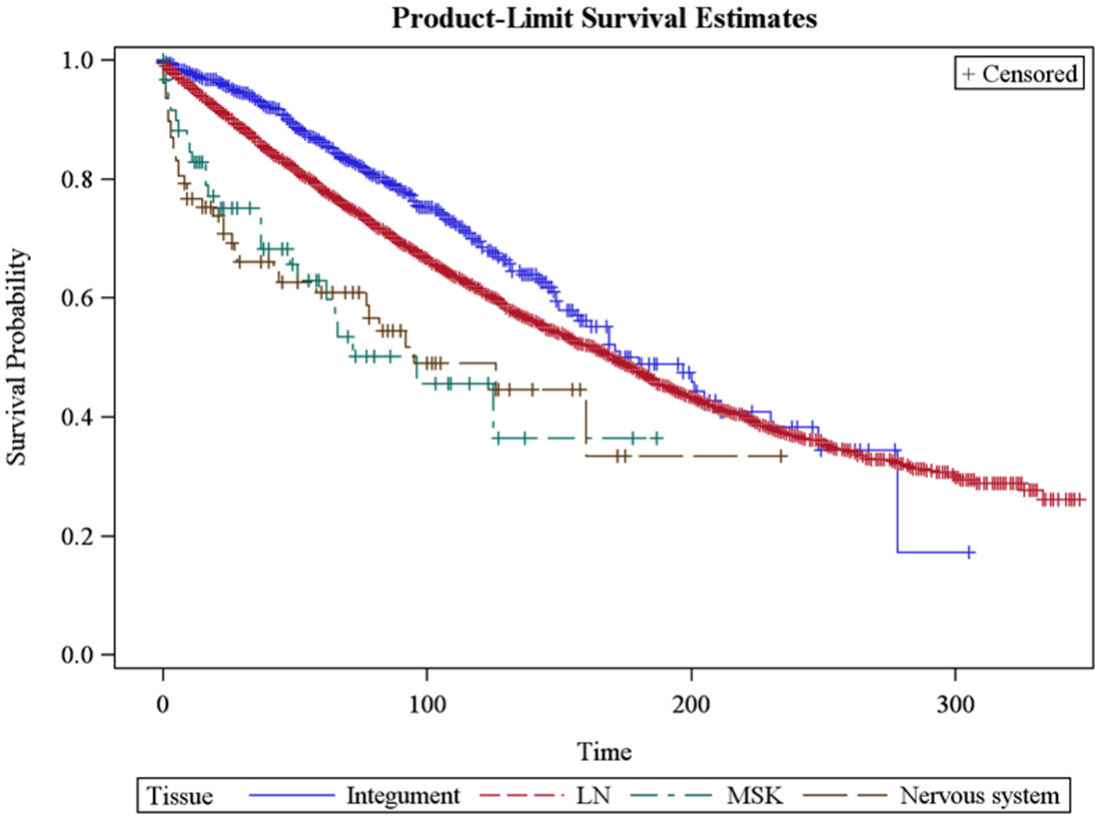
Kaplan-Meier curve demonstrating overall survival of patients with stage I follicular lymphoma **(A)** Lymph node primary site vs. integumentary system (median OS 170 vs. 180 months, p =0.001, blue line); **(B)** lymph node primary site vs. musculoskeletal system (median OS 170 months vs. 96 months, p =0.001, green hatched line); and **(C)** lymph node primary site vs. nervous system (median OS 170 vs. 95 months, p=0.01, brown hatched line). OS for other sites not statistically significant compared to lymph node primary site.

We performed a survival analysis comparing each primary extranodal site of disease with LN primary disease. On univariable analysis, integumentary disease was associated with better overall survival (p = 0.001, HR 0.74, 95% CI 0.63-0.86) when compared to a nodal primary site. Primary disease of the respiratory system was associated with worse OS (p = 0.037, HR: 1.68, 95% CI 1.18-2.4) as well as primary disease of the muscle & connective tissue system (p = 0.003, HR: 2.02, CI: 1.37-3; Table 2). On multivariable analysis, integumentary disease remained associated with better OS (p =0.001, HR 0.77, CI 0.66 to 0.9), while primary disease of the nervous system (p =0.01, HR 2.4, CI 1.72 to 3.38) and muscle & connective tissue system (p = 0.001, HR 2.14, CI 1.44 to 3.18) were associated with worse OS. Primary respiratory site was no longer significantly associated with survival on multivariate analysis (p =0.66, HR 1.39, CI 0.977 to 1.986; see also Table 2 and Figure 3). The other primary sites were not significantly associated with differential OS compared to LN primary site.

**Table 2:** Cox proportional hazards model showing overall survival, stratified by primary site

Primary site	Univariable analysis		Multivariable analysis	
	HR (95% CI)	P value	HR (95% CI)	P value
Integumentary System	0.74 (0.63-0.86)	0.001	0.77 (0.66-0.90)	0.001
Respiratory System	1.68 (1.18-2.4)	0.037	1.39 (0.97-1.99)	0.66
Musculoskeletal System	2.02 (1.37-3.0)	0.003	2.14 (1.44-3.18)	0.001
Nervous System	1.92 (1.37-2.69)	0.001	2.40 (1.72-3.38)	0.011

CI, confidence interval; HR, hazard ratio; RES, reticuloendothelial system, p-value adjusted by Bonferroni correction.

Patients treated in the pre-rituximab era had a worse OS on multivariate analysis than if treated in the rituximab era (p< 0.001, HR: 1.59, CI: 1.43-1.73). Female sex was associated with better OS (p< 0.001, HR: 0.76, CI: 0.71-0.81); increased age at diagnosis was associated with decreased survival and for every one-year increment in age, the risk of death increased by 7.1% (p<0.001, HR: 1.071, CI: 1.07-1.08). Patients who underwent treatment for their stage I FL with either surgery (p<0.001, HR: 1.58, CI: 1.46-1.71) or radiation (p<0.001, HR 1.36, CI 1.26-1.47) had better survival than those who did not receive these therapies. Race was not associated with OS.

## DISCUSSION

In a large, nationally representative, retrospective cohort study of patients with stage 1 FL, we observed that specific sites of involvement are associated with better or worse survival after adjusting for age, sex, race and therapy. Although patients with FL have an excellent median OS approaching 20 years in the modern chemotherapy era, a majority of patients continue to relapse within 5 years of their initial treatment. The treatments they receive are also associated with second malignancies and organ dysfunction [18-20].

Finding additional prognostic factors that supplement the FLIPI score will help refine it and risk stratify patients as having good versus poor risk. This in turn will guide therapeutic decision-making that will improve the quality of life for good risk patients and decrease the impact of overtreatment and the accompanying financial toxicity of cancer treatment.

Our findings demonstrate that stage 1 FL of the integumentary system has a significantly better outcome than LN primary disease. Primary cutaneous follicular lymphoma (PCFL) has traditionally been associated with an improved survival and response to therapy in prior studies [21, 22]. It is also designated as a specific category in the WHO classification [23]. These findings suggest that Stage I FL of the integumentary system is pathobiologically a different disease than LN primary disease. This may be due to the previously described rare expression of BCL-2 and a different gene signature in comparison to nodal follicular lymphoma [24-27]. In comparison, stage I FL of the muscle & connective tissue system and nervous system have a significantly worse survival than LN primary disease while other disease sites like the GI tract, head & neck, and respiratory system are not significantly associated with worse survival on multivariate analysis. Muscle & connective tissue disease being anatomically closer to bone, may represent transformed FL and this could explain the poor outcomes. A case series in the literature suggest that nervous system involvement by NHL carries a very poor prognosis with overall survival approximated at 4 months [28]. Although most cases of primary CNS lymphoma reported in the literature are diffuse large B cell lymphomas, a minority of cases has been pathologically confirmed to be follicular lymphoma. These cases retained the poor prognosis similarly to what we observed in our study [29]. The integumentary system and GI tract were the most commonly involved extranodal site of disease as previously reported [30]. Otherwise only limited data on the prognostic significance of non-cutaneous extranodal sites of FL exists which limits deriving definite conclusions on their impact on survival [31, 32].

Multiple epidemiological studies conducted have indicated that NHL characteristics, incidence and survival rates are influenced by race. This is less clear for FL probably due to the long indolent course of the disease and good overall survival [18, 33-37]. In our study we found no association of race on overall survival for stage I FL. FL is much more common in Whites than in other races [38]. We similarly found a much smaller representation of Black and other races (10%) in comparison to Whites (90%), which may further limit statistical power to detect a small survival differences based on race. Older age and male sex predicted for inferior patient survival in our study as previously reported [6, 10].

The use of radiation therapy in early stage FL has been associated with improved OS after correcting for socio-demographic, tumor and treatment factors [10, 39]. Involved-field RT is a commonly used treatment modality for early-stage FL [40]. Rituximab has also emerged as an efficient systemic therapy, though clinical data are limited regarding its impact in early stage FL. Although radiation therapy is considered to be better tolerated than chemoimmunotherapy, its use in early stage FL continues to decline in favor of alternative treatment strategies of single agent chemotherapy or observation [39]. We found a beneficial effect on OS when patients were initially treated with radiation therapy for their stage I FL. Our observation is in contrast to the general paradigm that the watch & wait approach is equivalent to the early treatment approach and supports findings from a growing body of literature that has shown improvement in quality of life indices as well as improvement in OS with the early treatment approach, making a case for carefully selecting the patient population that can benefit from this strategy [39, 41, 42].

The SEER database represents 28% of the United States population and provides a real world, multi-ethnic setting to further study the association between specific sites of involvement by follicular lymphoma, its clinical characteristics and outcomes. Our study is the largest to date to address this question for stage 1 follicular lymphoma.

However, a limitation of the SEER database is the lack of information about chemotherapy in the SEER database as a major modality of treatment for FL is chemoimmunotherapy. We partially addressed this issue by analyzing survival in pre-rituximab and rituximab era and in keeping with previously reported literature; we found an improved survival in the rituximab era [43]. Information about staging modalities is also limited. Staging patients accurately is the cornerstone of distinguishing early stage disease from disseminated disease. FDG-PET upstages a significant proportion of patients compared to conventional CT scans and aids in patients receiving the appropriate stage specific therapy and might not have been available for the majority of patients included in this analysis [10]. It is also unclear how many patients underwent a bone marrow biopsy as part of their staging work up. Incorporating information about staging modalities used in future studies will prove useful as the Lymphocare study reported that adequately staged patients had a better outcome regardless of the modality of treatment selected [10]. Another drawback is the lack of central pathologic confirmation [44]. Studies including patients diagnosed before 2001 had codes from earlier ICD-O versions that were converted to ICD-O-3 and have higher proportions of unclassified (e.g., lymphoma and not otherwise specified) cases. However, Clarke et al showed an 89% and 84% agreement between computer-converted ICD-O-2 codes to ICDO-3 codes and registry-assigned codes for follicular lymphoma cases diagnosed in the 1988-1994 and 1998- 2000 SEER periods respectively [45].

## MATERIALS AND METHODS

### Patients

We obtained data from the Surveillance, Epidemiology & End Results (SEER) program. SEER collects cancer incidence, treatment, and survival information from 18 geographic areas in the United States, representing 28% of the entire US population [46, 47]. We used direct case listings extracted by SEER*Stat software *version 8.1.5, released March 31,2014.* This report includes all FL patients in the SEER database during 1/1/83 and 12/31/11.

### Measurements

We identified patients with a diagnosis of FL using the International Classification of Disease for Oncology, 3^rd^ edition, ICD-0-3 histology code 9690, 9691, 9695, 9698 until the latest follow-up recorded in the SEER submission. These codes have been validated in previous studies [44, 48]. SEER identifies cases as follicular lymphoma based on several parameters that include histopathology, FISH testing, genetic testing for the BCL2 gene rearrangement, translocation t (14, 18) (q32;q21) and immunophenotyping. Our inclusion criteria were Stage I FL patients, age > 18 years, patients with active follow-up, and those with a single primary tumor. We excluded patients with a diagnosis established by autopsy and/or death certificate only, patients for whom the diagnosis of FL was a second or subsequent primary, and patients with an unknown primary site.

Primary sites were classified by organ or anatomic site into 1) head and neck 2) gastrointestinal (GI tract) 3) pulmonary system 4) thymus, mediastinum, and heart 5) muscle & connective tissue 6) integumentary system 7) nervous system 8) breast tissue 9) genitourinary system 10) endocrine system and 11) lymphatic system. PCFL and stage I FL of the integumentary system represent the same entity, however prior to 2008, PCFL was not recognized and as such not reported as a separate entity [49, 50]. Hence, for ease of understanding, we refer to this category as the integumentary system throughout the paper. We excluded the category of blood and reticuloendothelial system as this includes disease in the bone marrow, which cannot be easily distinguished from more advanced stages of disease. We subsequently defined a pre-rituximab era (1983-1998) and the rituximab era (1999-2011) based on the year of FDA approval of rituximab for the treatment of FL in the US to indirectly estimate the effect of rituximab on survival.

### Statistical analysis

We calculated OS as time in months from date of diagnosis and date of death, date last known to be alive, or the date of study cutoff. We performed descriptive statistics with Pearson’s Chi-square test to analyze categorical variables. For continuous variables we used Mann Whitney or Student t tests, depending on the normality of distribution of data. OS estimates were calculated using Kaplan-Meier survival analysis. Multivariable Cox proportional-hazard regression models were fitted to evaluate the prognostic impact of each extranodal site of involvement using the lymph node group as reference, after adjustment for known prognostic factors such as age, sex, race, surgery, and radiation. All tests were two-tailed, with the threshold for significance of p value set at 0.05. The p-values were adjusted by the Bonferroni correction. All statistical analyses were performed using SAS, *version 9.3.*

## CONCLUSION

In summary, primary site of disease is an important prognostic factor for patients with early stage FL as demonstrated by our population-based study. Patients with Stage I FL of the integumentary system had a significantly better outcome than primary nodal disease. Musculoskeletal and nervous system primary sites had a significantly worse survival than primary nodal sites. Furthermore, RT and surgery were associated with better survival than other treatment modalities, including expectant observation. This supports the hypothesis that a subset of patients with stage I FL may benefit from early and/or aggressive treatment in comparison to an observation only approach. It would be important to prospectively validate this finding in the current era of more accurate initial staging and more effective therapy that includes monoclonal CD20-directed antibodies.

In addition to its prognostic significance, primary site may correlate with certain biological characteristics associated with disease behavior and pathogenesis. Going forward it will be important to elucidate different pathobiological characteristics that may be specific to site of involvement by comprehensive genomic and mutational analysis. This might help identify pathways that can be therapeutically targeted with novel agents with low toxicity.
